# New and Recent Results for Thermoelectric Energy Conversion in Graded Alloys at Nanoscale

**DOI:** 10.3390/nano12142378

**Published:** 2022-07-12

**Authors:** Vito Antonio Cimmelli, Patrizia Rogolino

**Affiliations:** 1 Department of Mathematics, Computer Science and Economics, University of Basilicata, Viale dell’Ateneo Lucano, 10, 85100 Potenza, Italy; 2 Department of Mathematical and Computer Sciences, Physical Sciences and Earth Sciences, University of Messina, Viale F. Stagno d’Alcontres, 31, 98166 Messina, Italy; progolino@unime.it

**Keywords:** graded Silicon–Germanium alloys, efficiency of a thermoelectric process, figure-of-merit, entropy production, minimum of energy dissipated

## Abstract

In this article, we review the main features of nonlocal and nonlinear heat transport in nanosystems and analyze some celebrated differential equations which describe this phenomenon. Then, we present a new heat-transport equation arising within the so-called thermomass theory of heat conduction. We illustrate how such a theory can be applied to the analysis of the efficiency of a thermoelectric energy generator constituted by a Silicon–Germanium alloy, as the application and new results for a nanowire of length L=100 nm, are presented as well. The thermal conductivity of the nanowire as a function of composition and temperature is determined in light of the experimental data. Additionally, the best-fit curve is obtained. The dependency of the thermoelectric efficiency of the system on both the composition and the difference of temperature applied to its ends is investigated. For the temperatures T=300 K, T=400 K, and T=500 K, we calculate the values of the composition corresponding to the optimal efficiency, as well as the optimal values of the thermal conductivity. Finally, these new results are compared with recent ones obtained for a system of length L=3 mm, in order to point out the benefits due to the miniaturization in thermoelectric energy conversion.

## 1. Modeling Nonlocal and Nonlinear Heat Transport

Heat transport theory is currently broadening its field of applicability since, owing to the miniaturization, new phenomenologies, beyond the classical Fourier theory of heat conduction, have been discovered [1,2]. Those new phenomena depend on the relationship between the mean free path of the heat carriers *ℓ*, and the characteristic dimension of the conductor *L*, expressed by the Knudsen number Kn=ℓ/L. Fourier’s law is valid when ℓ/L≪1, namely, for ℓ≪L. However, Kn can increase for a reduction of *L*, as in miniaturization technologies.

One of the issues needing to be rediscussed when Kn≃1 is the concept of temperature and its relationship to heat transport [3,4,5].

A second open problem is the form of the heat-transport equation, since, when the mean free path of the heat carriers is comparable to the characteristic dimension of the conductor, i.e., Kn≃1, more complicated transport laws for the heat flux are necessary. Furthermore, if generalized heat transport equations are used, it appears that the analysis of their coherence with the second law of thermodynamics requires a generalized mathematical framework [6,7,8].

Nowadays, there is a current interest in mesoscopic approaches based on simpler generalized heat-transport equations than the much more complex and detailed microscopic one. Examples of these approaches are phonon hydrodynamics [4,5], and thermomass theory [9,10]. All of these models consider heat carriers as a fluid, whose hydrodynamic equations of motion describe the transport of heat.

The phonon hydrodynamics leads to the Guyer–Krumhansl transport equation [11,12,13,14] for the heat flux q, i.e.,

(1) $$\tau \frac{\partial q}{\partial t}+q=-\lambda \nabla T+\ell^{2}\left(\nabla^{2}q+2\nabla \nabla \cdot q\right),$$

where τ is the relaxation time due to the resistive (quasi-momentum not conserved) scattering of phonons in the bulk, and *T* is the temperature. Moreover, the constant $\lambda =\varrho c_{v}\tau {\bar{v}}^{2}/3$, where ϱ is the mass density, $c_{v}$ the specific heat per unit mass at constant volume, and $\bar{v}$ is the average of the phonons’ speed, is the thermal conductivity.

Whenever nonlocal effects are negligible (namely, $\ell^{2}≃0$), Equation (1) reduces to the Maxwell–Cattaneo–Vernotte equation [15]

(2) $$\tau \frac{\partial q}{\partial t}+q=-\lambda \nabla T,$$

which describes heat conduction with finite speed [16]. Equation (2), in turn, generalizes the classical Fourier equation

(3) q=−λ∇T,

by including relaxation effects through the term $\tau \frac{\partial q}{\partial t}$.

Equations (1) and (2) do not take into account the non-linear effects, which instead are common at the micro/nanoscale. Extensions of Equations (1) and (2) to the non-linear regime can be obtained within the frame of Extended Irreversible Thermodynamics (EIT) [4,5,6,14,17,18]. For instance, the nonlinear Guyer–Krumhansl equation takes the form

(4) $$\tau \frac{\partial q}{\partial t}+q=-\lambda \nabla T+\mu q\cdot \nabla q+\ell^{2}\left(\nabla^{2}q+2\nabla \nabla \cdot q\right),$$

where $\mu =2\tau /c_{v}T$. When nonlocal effects can be neglected (ℓ≃0), Equation (4) yields a nonlinear generalization of the Maxwell–Cattaneo Equation (2).

A further nonlinear generalization of Equation (2) may be obtained within the framework of the thermomass (TM) theory [9,10]. In the latter, such an equation reads [9,19,20]

(5) $$\tau_{\mathrm{tm}}\frac{\partial q}{\partial t}-\rho c_{v}\frac{\partial T}{\partial t}l+\nabla q\cdot l+\lambda \left(1-b\right)\nabla T+q=0,$$

wherein $\tau_{\mathrm{tm}}$ is a relaxation time [19,20,21], *b*, (<1), is a dimensionless physical parameter entering the effective thermal conductivity $\lambda_{eff}\equiv \lambda \left(1-b\right)$, and l denotes a characteristic-length vector [20,21]. In thermomass description, the heat flux is generated a gas of heat carriers, characterized by an effective mass density and flowing through the medium under the action of a thermomass-pressure gradient. This gas is made by massive quasi-particles of heat carriers, named thermons, which are nothing but the vibrations of the molecules generated by heating the conductor, with null rest-mass and dynamic mass which may be calculated from the Einstein’s mass-energy duality. In gases and liquids, the thermons are supposed to be attached to the molecules or atoms of the medium. In solids, the thermomass gas coincides with the phonon gas for crystals, attached on the electron gas for pure metals, or just both of them for systems in which the heat carriers are phonons and electrons. The physical parameters entering Equation (5) are

$$\tau_{\mathrm{tm}}=\frac{\lambda }{2\gamma \rho c_{v}^{2}T}\;\;,$$

with the dimensionless parameter γ being the Grüneisen constant,

$$b=\frac{q^{2}}{2\gamma \rho^{2}{\left(c_{v}T\right)}^{3}}\;\;,$$

stands for a dimensionless number which is called thermal Mach number of the drift velocity relative to the thermal-wave speed in the heat-carrier collection, and

$$l=\frac{\lambda q}{2\gamma \rho c_{v}{\left(c_{v}T\right)}^{2}}\;\;,$$

is a characteristic-length vector. In fact, the physical dimensions of |l| are meters, as it can be directly inferred by the dimensional analysis of Equation (5). It characterizes the strength of the non-Fourier effects introduced by Equation (5) and, for conceivable values of q, attains values which are always much smaller than those of the mean-free path of the thermons [20,21].

Both Equations (4) and (5) account for nonlinear and nonlocal effects, the sole difference being that Equation (5) is capable of describing only first-order nonlocality through the term ∇q·l, while Equation (4) accounts for first-order nonlocality, through the term μq·∇q, and second-order nonlocality, through the term $\ell^{2}\left(\nabla^{2}q+2\nabla \nabla \cdot q\right)$.

In our analysis, we will apply Equation (5), since it contains the meaningful concept of effective thermal conductivity, i.e., the experimental evidence that the thermal conductivity is not independent of the heat flux. We will see that such a conductivity will influence in a meaningful way the behavior of the systems under investigation. The results followed by Equation (4) in comparison with the present ones, will be the subject of future research.

## 2. Thermoelectric Energy Conversion at Nanoscale

In the last decades, nanosystems have been widely applied in modern technology. The word nanosystem means systems with at least one dimension at the nanoscale. They provide an interesting avenue to obtain highly performing devices, for example, making nanocomposites, adding nanoparticles to a bulk material, or using one-dimensional nanostructures. Currently, the research on nanotechnology involves the preparation of different types of nanomaterials and analysis of their properties for applications in medical technology, microelectronics, aerospace, energy production and management, and biotechnology. The main reason of such a wide field of application is that recent industrial techniques allow for modifying the properties of nanomaterials in order to adapt them for utilizing in several applications [22]. For instance, due to their low impedance and strong chemical inertia, metal nanoparticles have a wide application to enhance the performance of microelectronic devices [23]. Meaningful advances in thermal transport in nanocarbon assemblies have been obtained as well [24].

One of the most important applications of nanosystems is the thermoelectric energy conversion. Thermoelectric effects are generated by the intrinsic relation between electric and thermal properties of a system. The two primary thermoelectric effects are the Seebeck effect and the Peltier one. The Seebeck effect describes the creation of an electric current by a temperature difference, while the Peltier effect describes the generation of a heat flux by a current circulation [18,25]. Thermoelectric devices as energy converters are easily produced and do not have moving parts, or liquid fuels. Moreover, unlike traditional heat engines, thermoelectric generators are completely silent, and do not produce pollution. Thus, several current studies are devoted in finding new materials with appropriate properties, allowing the production of efficient thermoelectric devices. To achieve that task, one of the most explored possibilities is the use of some nanostructured materials, allowing one to enhance the thermodynamic efficiency of the devices. Indeed, a good thermoelectric material should have very low thermal conductivity, but a very high electrical conductivity, and such properties are easier to obtain at nanoscale. For instance, in semiconductors, the reduced dimension can produce an increment of the phonon scattering, and a consequent reduction of the thermal conductivity. Thus, the efficiency of thermoelectric devices can be enhanced with the use of nanotechnology. Carbon nanotubes and graphene sheets are excellent examples of thermoelectric materials. Moreover, nowadays one-dimensional devices, such as nanowires, have considerably attracted the attention of the researchers, in virtue of their excellent thermoelectric properties.

The two main fabrication techniques of nanowires are the top-down fabrication and the bottom-up synthesis. The first one is a subtractive technique, such as the creation of a sculpture from an original block of stone. The second one is an additive technique, such as the growth of a pearl from a small grain of sand. A typical top-down fabrication method is the lithography, and a typical bottom-up synthesis method is the vapor phase [26].

The thermoelectric energy converters considered here are rigid nanowires of length *L*. They will be either homogeneous, i.e., with a composition that is independent of the points of the nanowire, or with a composition varying along the nanowire. In the latter case, we consider a Silicon–Germanium alloy, which will be denoted by $Si_{c}Ge_{1-c}$, with the stoichiometric variable *c* dependent of the position *z* of the points of the system, and such that c(0)=0 and c(L)=1. Thus, for z=0 we have pure Germanium, and for z=L we have pure Silicon. In the intermediate points, namely for z∈[0,L], the composition *c* will depend on the function c(z), which is determined while manufacturing the material. The most easy situation is the linear dependency on *z*, namely c=z/L. For very small length *L*, for instance L=100 nm, such a system can be considered as representative of a Germanium–Silicon junction.

It is worth noticing that graded materials of the type $A_{c}B_{1-c}$, with *A* and *B* two different species, and composition *c* changing along the length of the system, have many practical applications in heat transfer problems, especially at a micro/nanoscale. One of them is the heat rectification, namely, the fact that the same temperature gradient but acting in opposite directions on a given system yields different values of the corresponding heat flux [27].

**Figure 1:**
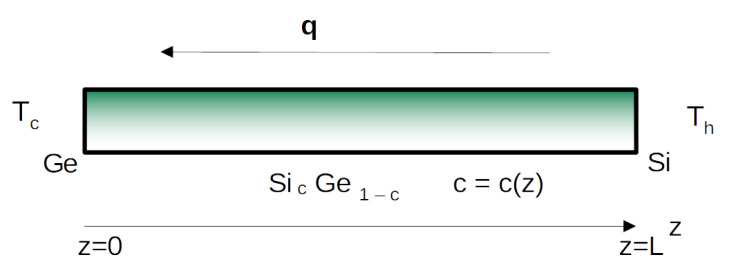
Sketch of a $Si_{c}Ge_{1-c}$ nanowire, of length *L*, with the difference of temperature $T_{h}-T_{c}$ at its ends.

In Figure 1, a $Si_{c}Ge_{1-c}$ nanowire, of length *L*, with the difference of temperatures $T_{h}-T_{c}$ at its ends, is schematized.

The efficiency of a thermoelectric system is defined as

(6) $$\eta =\frac{P_{\mathrm{el}}}{{\dot{Q}}_{\mathrm{tot}}},$$

with $P_{\mathrm{el}}$ the obtained electric power, and ${\dot{Q}}_{\mathrm{tot}}$ the total heat per unit time entering the system. If one remains in the frame of linear thermodynamics, i.e., with linear constitutive equations for heat flux and electrical current, it can be proven that for a nanosystem whose two sides are constantly maintained at different temperatures $T_{h}$ (the hottest temperature) and $T_{c}$ (the coldest one), the maximum efficiency is [25]

(7) $$\eta_{max}=\eta_{C}\frac{1-1/\xi }{1+1/\xi },$$

wherein $\eta_{C}=1-T_{c}/T_{h}$ is the classical Carnot efficiency, $\xi \equiv \sqrt{ZT+1}$, and *Z*, the most important physical parameter in thermoelectricity is the so-called figure-of-merit. Its expression is $Z=\frac{\epsilon^{2}\sigma_{e}}{\lambda }$, with ϵ as the Seebeck coefficient, $\sigma_{e}$ as the electrical conductivity, and λ as the thermal conductivity [25]. Such a result is in accordance with the general tenets of finite time thermodynamics [28,29], always with $\eta_{max}<\eta_{C}$. Thus, since the higher ξ, the higher $\eta_{max}$, several studies in the last decades focused on the methods to enhance ξ, i.e., to enhance ZT. In nanosystems such a goal can be achieved, for instance, by searching the strategies to reduce λ. However, this is not a easy task, since a reduction of λ would increase the phonon scattering [14,18], and this produces dissipation which, in turn, reduces $P_{\mathrm{el}}$. Thus, the numerator and denominator in Equation (6) cannot be controlled independently. On the other hand, to optimize only one of them is not sufficient, as argued by Hoffmann in [29], where it is shown by a counterexample that the maximum of $P_{\mathrm{el}}$ does not correspond to the maximum of η.

The aim of this article is twofold.

For homogeneous nanowires, which are analyzed in Section 3, we review the corrections, derived in [30], to the classical evaluation of η, once nonlocal and nonlinear effects are taken into account. Such a calculation is necessary, because in classical electricity such effects are not taken into account [25], and hence, using the formulae of classical thermoelectricity would introduce errors in the estimation of thermoelectric efficiency at nanoscale.

For graded nanowires, we determine the physical conditions yielding the best value of η, in such a way that the efficiency of the thermoelectric system is optimal. Such information is important in energy conversion and management.

To this end, we apply a new approach, which does not maximize η but minimizes the rate of energy dissipated along the process. In this way, we overcome the problems arising in maximizing η. Then, in Section 4, we review the mathematical procedure for minimizing the energy dissipated, while in Section 5, we review the procedure applied to obtain the best fit of the thermal conductivity as a function of the composition, by the experimental data at our disposal. We have chosen the three temperatures T=300 K, T=400 K and T=500 K because in this way we cover a wide range of possible operational temperatures. For the three cases analyzed here, the final results are rather close, so that we expect that similar results can be obtained for intermediate temperatures. In Section 5, the procedure is applied, for the first time, to a nanowire of length L=100 nm, not yet considered in previous investigations. Those new results show remarkable differences with previous results obtained for a macroscopic one dimensional system of length L=3 mm. A comparison is made in Section 6, wherein it is inferred that the miniaturization of thermoelectric systems allows one to improve their efficiency through a reduction of their thermal conductivity.

## 3. Influence of Nonlocal and Nonlinear Effects on the Efficiency of Thermoelectric Systems

In this section, we point out that, for thermoelectric systems whose constitutive equation for the heat flux allows one to account for nonlocal and nonlinear effects, the efficiency is remarkably different with respect to that calculated by using the classical Fourier law [25]. To this end, let us consider a single thermoelectric generator of length *L*, the two sides of which are steadily kept at the different temperatures $T_{h}$ (the hotter temperature) and $T_{c}$ (the colder one), in such a way that both a quantity of heat per unit time uniformly flows through the system, and an uniform electric current is produced by the Seebeck effect. In such a case, the heat flux given by the constitutive equation

(8) q=−λ∇T+Πi,

wherein Π is the Peltier coefficient, and i denotes the electric-current density. The right-hand side of Equation (8) consists in the classical term proportional to the gradient of temperature (Fourier law), plus the additional heat flux Πi generated by the current circulation. The constitutive equation for the current density is

(9) $$i=-\sigma_{e}\epsilon \nabla T+\sigma_{e}E,$$

with E as the electric field, herein regarded as an external force applied to the system [25].

Finally, Equations (8) and (9) must be coupled with the energy-rate equation

(10) $$\rho \frac{\partial u}{\partial t}=-\nabla \cdot q+E\cdot i,$$

where *u* is the specific internal energy, and the quantity E·i is the rate of energy production due to the circulation of the electric current.

For such a system, the energy dissipated along the process locally can be rewritten as

(11) $$T\sigma_{s}=E\cdot i-\left(\frac{\nabla T}{T}\right)\cdot q\;\;\;,$$

where $\sigma_{s}$ denotes the local entropy production. The second law of thermodynamics imposes that the left-hand side of Equation (11) must be non-negative. According to the classical Onsager approach [31], the right-hand side of Equation (11) can be regarded as the product of thermodynamic forces and fluxes. Then, it is always non-negative provided the following linear relations hold

(12a) $$\begin{array}{cc} \; & q=-L_{11}\frac{\nabla T}{T}+L_{12}E, \end{array}$$

(12b) $$\begin{array}{cc} \; & i=-L_{21}\frac{\nabla T}{T}+L_{22}E, \end{array}$$

with the matrix of the phenomenological coefficients $L_{\mathrm{ij}},\;\;\left(i,j=1,2\right)$, positive semi-definite.

This yields

(13a) $$\begin{array}{cc} \; & L_{11}=\left(\lambda +\Pi \sigma_{e}\epsilon \right)T, \end{array}$$

(13b) $$\begin{array}{cc} \; & L_{12}=\Pi \sigma_{e},\;\;\; \end{array}$$

(13c) $$\begin{array}{cc} \; & L_{21}=\sigma_{e}\epsilon T,\;\;\; \end{array}$$

(13d) $$\begin{array}{cc} \; & L_{22}=\sigma_{e}.\;\;\;\; \end{array}$$

Then, by the Onsager Symmetry Relation (OSR) $L_{12}=L_{21}$, [31], the celebrated Second Kelvin Relation (SKR) Π=ϵT ensues.

On the other hand, if we take into account Equations (6), (8) and (9), making use of the approximation ∇T≃ΔT/L, and of the SKR, we obtain

(14) $$\eta =\eta_{C}\left[\frac{\epsilon y-y^{2}\lambda \sigma_{e}^{-1}}{T_{h}^{-1}+\epsilon y}\right],$$

wherein $y=\frac{iL}{\lambda \left(T_{h}-T_{c}\right)}$.

The right-hand side of Equation (14) is the expression of thermoelectric efficiency in the linear regime. In the nonlinear regime, according to thermomass theory, the heat flux transforms in

(15) $$q=-\nabla q\cdot l-\lambda \left(1-b\right)\nabla T+\Pi i.$$

Then, under the hypotheses that both q and E take a constant value in any transversal section along the longitudinal axis, and that q and i are parallel, we obtain

(16) $$q=-\left[\lambda \left(1-b\right)+\sigma_{e}\epsilon \left(\Pi -\bar{E}\;\bar{l}\right)\right]\nabla T+\sigma_{e}\left(\Pi -\bar{E}\;\bar{l}\right)E,$$

where $\bar{E}$ and $\bar{l}$ denote the mean values of |E| and |l| on the interval [0,L] [30].

Thus, by comparison of Equations (12a) and (12b), with Equations (16) and (9) respectively, we obtain

(17a) $$\begin{array}{cc} \; & L_{11}=\lambda \left(1-b\right)T+\sigma_{e}\epsilon \left(\Pi -\bar{E}\;\bar{l}\right)T, \end{array}$$

(17b) $$\begin{array}{cc} \; & L_{12}=\sigma_{e}\left(\Pi -\bar{E}\;\bar{l}\right),\;\;\;\;\;\; \end{array}$$

(17c) $$\begin{array}{cc} \; & L_{21}=\sigma_{e}\epsilon T,\;\;\;\;\;\;\;\;\; \end{array}$$

(17d) $$\begin{array}{cc} \; & L_{22}=\sigma_{e}.\;\;\;\;\;\;\;\;\;\; \end{array}$$

By Equations (17b) and (17c) we infer that the following different conditions may occur [30].

1. The OSR holds and the SKR is not valid.
  In such a case, from Equations (17b) and (17c) it follows
  (18) $$\Pi =\epsilon T+\bar{E}\;\bar{l},$$
  so that the SKR is no longer valid. In this case, due to the contribution of the nonlinear terms in the constitutive equation of the heat flux, the following thermoelectric efficiency ensues
  (19) $$\eta =\eta_{C}\left[\frac{\epsilon y-y^{2}\lambda \sigma_{e}^{-1}}{\left(1-b\right)T_{h}^{-1}+\epsilon y}\right],$$
  once Equation (18) has been taken into account [30]. From a comparison of the above expression with Equation (14) it can be deduced that η is influenced by the nonlinear term λb∇T, which leads to an increment of η, depending, through *b*, on the intensity of the heat flux [30].
2. The SKR holds and the OSR is not valid.
  If, instead, the SKR holds, then the thermoelectric efficiency becomes
  (20) $$\eta =\eta_{C}\left[\frac{\epsilon y-y^{2}\lambda \sigma_{e}^{-1}}{\left(1-b\right)T_{h}^{-1}+\left(\epsilon -\bar{E}\;\bar{l}T_{h}^{-1}\right)y}\right].$$
  Thus, an increment in η is achieved by the reduction of the thermal conductivity $\lambda \left(1-b\right)$, and by the presence of the second nonlinear term ∇q·l [30].
3. Both the OSR and the SKR break down.
  In such a situation, the thermoelectric efficiency becomes
  (21) $$\eta =\eta_{C}\left[\frac{\epsilon y-y^{2}\lambda \sigma_{e}^{-1}}{\left(1-b\right)T_{h}^{-1}+\left(\Pi -\bar{E}\;\bar{l}\right)T_{h}^{-1}y}\right],$$
  which again is influenced by both nonlinear terms. However, since no relation between Π and ϵT holds, we cannot say whether there is an improvement of η or not [30].

The considerations above demonstrate that for miniaturized systems, for which a nonlinear constitutive equation for the heat flux holds, the classical expression of the thermodynamic efficiency given by Equation (14) leads to an incorrect evaluation of η. One should use one of the three Equations (19)–(21), and which of them should be applied depends on the experimental conditions leading to the validity of either OSR, or SKR, or none of them.

## 4. Minimal Energy Dissipated and Efficiency of Thermoelectric Systems

The analysis of the efficiency of thermodynamic engines is based on the concept of the Carnot cycle, which is a quasi-static transformation consisting of two isothermal and two adiabatic arcs in the space state. The efficiency of a heat engine undergoing a Carnot cycle between the two temperatures $T_{c}$ (cold temperature) and $T_{h}$ (hot temperature), takes the form $\eta_{C}=1-T_{c}/T_{h}$. Since a quasi-static transformation takes over in an infinite time, $\eta_{C}$ is different from the efficiency of those processes which take over in a finite time. For these processes, the ratio $\eta =P_{\mathrm{ex}}/{\dot{Q}}_{\mathrm{tot}}$, seems to be more appropriate. So, for real trials, it is important to investigate how much $\eta \neq \eta_{C}$. In our investigations, we assume that in a thermoelectric process the Joule effect, at each point *z* and at any time *t*, induces a rate of energy dissipated E(z,t). Our basic postulate is that the best efficiency is achieved when E(z,t) is minimum. In fact, in a thermoelectric process of duration τ, the total dissipated energy is given by

(22) $$E_{tot}=\int_{0}^{L}\int_{0}^{\tau }E\left(z,t\right)dzdt.$$

On the other hand, the integrand being positive is tantamount to suppose that E(z,t) is at the minimum at any point in the integration domain.

We assume that a given quantity of heat per unit time ${\dot{Q}}_{\mathrm{tot}}$, as well as an electric current i, enters uniformly into the hot side of the generator.

Under such hypothesis, we obtain [30]

(23) $$E=\frac{i^{2}}{\sigma_{e}}+i\left[\epsilon \;T_{h}-\left(\Pi -\bar{E}\;\;\bar{l}\right)\right]\nabla T+\lambda \left(1-b\right){\left(\nabla T\right)}^{2}.$$

In what follows, we derive the conditions under which the efficiency of the system under consideration is at the maximum, by looking for the minima of the right-hand side of Equation (23), which depends on the temperature gradient as well as on the composition *c*. For the sake of simplicity, we make the approximation $\nabla T≃\frac{T_{h}-T_{c}}{L}$ and put

(24) $$x\equiv \sqrt{\frac{T_{h}-T_{c}}{L}}.$$

In this way, the right-hand side of Equation (23) takes the form

(25) $$E\left(c,x\right)=\frac{i^{2}}{\sigma_{e}}+i\left[\epsilon \;T_{h}-\left(\Pi -\bar{E}\;\;\bar{l}\right)\right]x^{2}+\lambda \left(c\right)\left(1-b\right)x^{4}.$$

Thus, we look for the eventual minima of the right-hand side of Equation (25).

It is worth observing that, from the physical point of view, the two variables *c* and *x* play a different role in the process of energy conversion. The first one can be determined only when the material is manufactured and it cannot be changed further after this phase, namely, when the finished product is completed. Th variable *x*, instead, can be tuned by the exterior during the process of energy production by tuning the temperature difference $T_{h}-T_{c}$. Thus, the efficiency of the finished nanowire is partially fixed and partially adaptable to the experimental needs.

The stationary points $\left(c_{opt},x_{opt}\right)$ of the function $E\left(c,x\right)$ above are given by the stationary points $c_{opt}$ of the function λ(c), and the values

(26) $$x_{opt}=\sqrt{\frac{-i\;[\epsilon \;T_{h}-\left(\Pi -\bar{E}\;\;\bar{l}\right)]}{4\;\lambda_{opt}\left(1-b\right)}},$$

wherein $\lambda_{opt}\equiv \lambda \left(c_{opt}\right)$. Those points exist if the inequality

(27) $$i\;[\epsilon \;T_{h}-\left(\Pi -\bar{E}\;\;\bar{l}\right)]<0,$$

is satisfied. Moreover, the function $E\left(c,x\right)$ admits a minimum if, and only if, the thermal conductivity has a minimum itself, and the further constraint

(28) $$2i\left[\epsilon \;T_{h}-\left(\Pi -\bar{E}\;\bar{l}\right)\right]+12\lambda_{opt}x_{opt}^{2}\left(1-b\right)>0,$$

is satisfied.

We note that, due to the constraint (27), the first term in the left-hand side of Equation (28) is negative. However the second term is positive, so that the constraints (27) and (28) are compatible.

## 5. Thermal Conductivity of Graded ${\mathrm{Si}}_{c}{\mathrm{Ge}}_{1-c}$ Alloys

Herein, we propose a model for the thermal conductivity of $Si_{c}Ge_{1-c}$ nanowires [32,33,34]. To this end, we apply a Non-Linear Regression Method (NLRM) [35,36], in order to obtain the best fit of the experimental data for the thermal conductivity of a wire of length L=100 nm as function of the composition. Based on the results of the experiments [37,38,39], we looked for a best-fit curve of the form

(29) $$\lambda \left(c\right)=\varphi \left(A,B,D,E\right)e^{Ac^{2}+Bc}+\vartheta \left(A,B,D,E\right)e^{Dc^{2}+Ec},$$

where *A*, *B*, *D*, and *E* are the unknown parameters and φ(A,B,D,E) and ϑ(A,B,D,E) are experimental coefficients [34]. We assign an initial estimated value of each parameter entering Equation (29), and then obtain the curve defined by the initial values of the parameters, in such a way that it is as close as possible to the experimental points. The conditions $\lambda \left(0\right)=\lambda_{Ge}$ and $\lambda \left(1\right)=\lambda_{Si}$ yield, finally,

$$\begin{array}{c} \varphi \left(A,B,D,E\right)=\frac{\lambda_{Si}-\lambda_{Ge}\;e^{D+E}}{e^{A+B}-e^{D+E}},\;\vartheta \left(A,B,D,E\right)=\frac{-\lambda_{Si}+\lambda_{Ge}\;e^{A+B}}{e^{A+B}-e^{D+E}}. \end{array}$$

**Figure 2:**
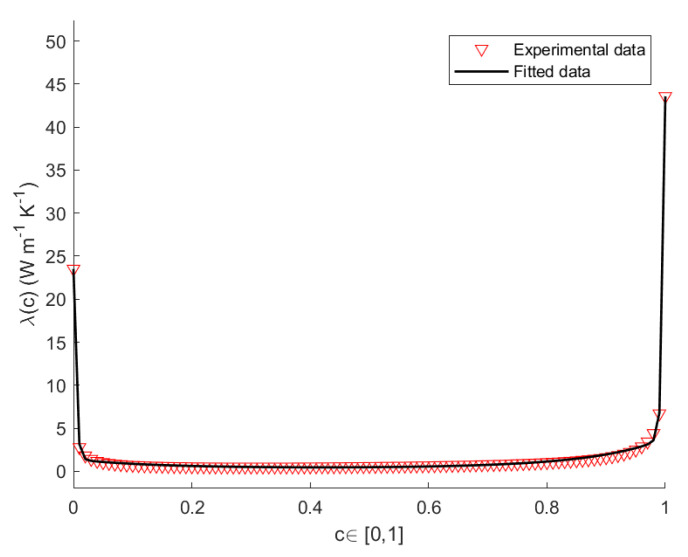
Theoretical and empirical λ(c) of the $Si_{c}Ge_{1-c}$ nanowire at T=300 K, for L=100 nm.

**Figure 3:**
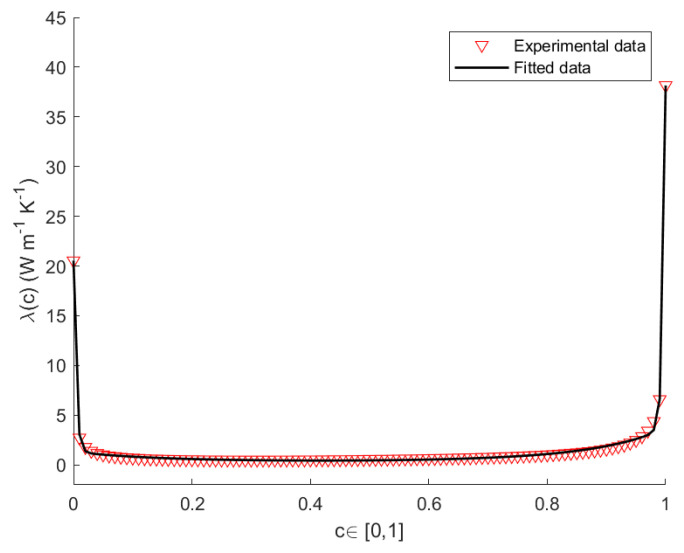
Theoretical and empirical λ(c) of the $Si_{c}Ge_{1-c}$ nanowire at T=400 K, for L=100 nm.

**Figure 4:**
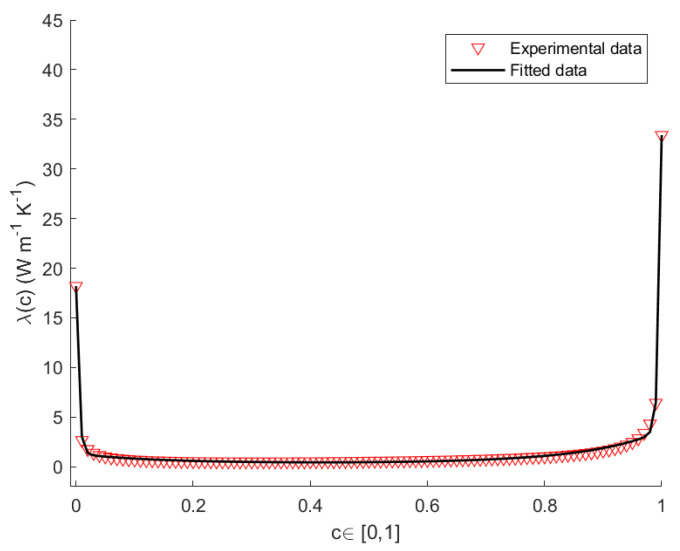
Theoretical and empirical λ(c) of the $Si_{c}Ge_{1-c}$ nanowire at T=500 K, for L=100 nm.

The values of the thermal conductivity for pure Si and pure Ge at T=300 K, T=400 K and T=500 K, are listed in Table 1. The values of *A*, *B*, *D*, *E* for a nanowire of length L=100 nm at the constant temperatures T=300 K, T=400 K, T=500 K, are listed in Table 2. Figure 2, Figure 3 and Figure 4 show the plots the experimental and theoretical functions of λ(c,T) given by Equation (29), at T=300 K, T=400 K and T=500 K, for L=100 nm. By direct inspection, it is evident in the results that the numerical error is very small, and that the fitted curves carefully reproduce the sequence of the experimental data.

## 6. Results and Discussion

In the present section, we analyze the minima $\left(c_{opt},x_{opt}\right)$ of $E\left(c,x\right)$, at different temperatures.

At T=300 K, the heat conductivity has its minimum value $\lambda =0.436925\;\;{\mathrm{Wm}}^{-1}K^{-1}$ for c=0.420249. In correspondence of this point $E\left(c,x\right)$ is at the minimum.

At T=400 K, the heat conductivity has its minimum value $\lambda =0.440330\;\;{\mathrm{Wm}}^{-1}K^{-1}$ for c=0.48914. In correspondence of this point $E\left(c,x\right)$ is at the minimum.

At T=500 K, the heat conductivity has its minimum value $\lambda =0.443582\;\;{\mathrm{Wm}}^{-1}K^{-1}$ for c=0.417490. In correspondence of this point $E\left(c,x\right)$ is at the minimum.

The previous results are summarized in Table 3.

The results here obtained seem to indicate that optimal efficiency takes place in the zone where λ is almost constant. For the sake of comparison, in Table 4 we show the values of $\lambda_{opt}$ and and $c_{opt}$ obtained in [34] for a wire of length L=3 mm. We note that the values of $\lambda_{opt}$ obtained therein are almost one order of magnitude higher with respect to those obtained here for L=100 nm. Thus, in light of the expression of *Z*, we conclude that miniaturization can improve the efficiency, since it reduces the value of the thermal conductivity. Such information constitutes a useful tool in manufacturing thermoelectric nanowires.

In our future investigations, we will consider the nonlinear extension of the Guyer–Krumhansl equation in Equation (4) [4].

Since Equation (4) also accounts for nonlocal effects through the second-order spatial derivatives of the heat flux, we aim at investigating whether such an equation leads to different results with respect to Equation (5).

## Figures and Tables

**Table 1:** Thermal conductivity (in $Wm^{-1}K^{-1}$) of pure Si and pure Ge at T=300 K, T=400 K, and T=500 K, for $Si_{c}Ge_{1-c}$ nanowires of length L=100 nm.

Temperature	$\lambda_{\mathrm{Si}}$	$\lambda_{\mathrm{Ge}}$
T=300 K	23.52	43.58
T=400 K	20.54	38.16
T=500 K	18.19	33.40

**Table 2:** Numerical parameters in Equation (29) for L=100 nm.

Temperature	A	B	D	E
T=300 K	6.380396	−5.362715	252.519739	−251.932542
T=400 K	6.305183	−5.282662	239.768796	−239.184401
T=500 K	228.240999	−227.674222	6.207791	−5.183391

**Table 3:** Values of $\lambda_{opt}$ (in ${\mathrm{Wm}}^{-1}K^{-1}$) and $c_{opt}$ at T=300 K, T=400 K and T=500 K, for L=100 nm.

Temperature	$c_{opt}$	$\lambda_{opt}$
T=300 K	0.420249	0.436925
T=400 K	0.418914	0.440330
T=500 K	0.417490	0.443582

**Table 4:** Values of $\lambda_{opt}$ (in ${\mathrm{Wm}}^{-1}K^{-1}$) and $c_{opt}$ at T=300 K, T=400 K and T=500 K, for L=3 mm.

Temperature (K)	$c_{\mathrm{opt}}$	$\lambda_{\mathrm{opt}}$ (in ${\mathrm{Wm}}^{-1}K^{-1}$)
T=300	0.385989	7.51235
T=400	0.375079	7.48291
T=500	0.36537	7.42273

## Data Availability

Not applicable.
